# Random Neuronal Networks show homeostatic regulation of global activity while showing persistent changes in specific connectivity paths to theta burst stimuli

**DOI:** 10.1038/s41598-018-34634-x

**Published:** 2018-11-08

**Authors:** Jude Baby George, Grace Mathew Abraham, Bharadwaj Amrutur, Sujit Kumar Sikdar

**Affiliations:** 1 Center for Nanosicence and Engineering, IISc Bangalore, Bangalore, India; 2 Robert Bosch Center for Cyber-Physical Systems and Department of Electrical Communications Engineering, IISc Bangalore, Bangalore, India; 3 Molecular Biophysics Unit, IISc Bangalore, Bangalore, India

## Abstract

Learning in neuronal networks based on Hebbian principle has been shown to lead to destabilizing effects. Mechanisms have been identified that maintain homeostasis in such networks. However, the way in which these two opposing forces operate to support learning while maintaining stability is an active area of research. In this study, using neuronal networks grown on multi electrode arrays, we show that theta burst stimuli lead to persistent changes in functional connectivity along specific paths while the network maintains a global homeostasis. Simultaneous observations of spontaneous activity and stimulus evoked responses over several hours with theta burst training stimuli shows that global activity of the network quantified from spontaneous activity, which is disturbed due to theta burst stimuli is restored by homeostatic mechanisms while stimulus evoked changes in specific connectivity paths retain a memory trace of the training.

## Introduction

Persistent changes in neuronal networks to recurrent stimuli have been thought of as the basis of learning and memory since the Hebbian principle was postulated^1^. Several experiments have demonstrated this phenomenon at synaptic level and different mechanisms have been identified for long term changes^2,3^. Theoretical studies and computer simulations show that mechanisms like LTP have a destabilizing effect on the overall network due to positive feedback as a result of increase in co-relations^1,4–6^. For networks to function effectively it is critical that mechanisms that allow for flexibility and those that maintain stability co-exist which together enable the organisms to maintain coherent responses while being adaptable^1^.

Neuronal networks have been shown to change their response to specific stimuli^7–9^. However the mechanisms by which the network can maintain its overall global properties while also being adaptable to new stimuli remain unclear^1^. Can the network be modified by a barrage of inputs like in a theta burst stimuli without a radical change in its activity? Destabilizing nature of such inputs has to be counterbalanced by some homeostatic mechanisms. Synaptic scaling has been demonstrated experimentally^10^. However, how these mechanisms operate at a functional level of the network to maintain stability over a range of spatial and temporal scales have not been studied. The experiments which simultaneously study persistent changes and homeostatic regulation are lacking.

The effect of such stimuli on neuronal networks has also been studied^11,12^. However, these studies do not discuss the persistent effect of such stimuli or investigate the process in detail. The integrated activity of individual neurons in a neuronal network determines the network output. The activity dependent changes in selective pathways at specific connections are affected by the complexity of the neuronal network and its global activity that is distributed throughout many neurons which in turn may modulate the connections selectively. Thus, the global activity that is distributed throughout many cells is equally important in determining the functional output of the system. The *in vitro* multi-electrode array (MEA) system provides a unique opportunity to simultaneously study fundamental mechanisms of global network function and plasticity and along specific connections^13^. Here we report that random neuronal networks have an inherent state-a reflection of global activity which affects the overall response of the network. We show that theta burst stimuli cause persistent changes at specific connectivity paths in the neuronal network. Interestingly, we find that such stimuli cause a temporary disturbance in the state of the network quantified from spontaneous activity that is restored in a short time while persistent changes at specific connections are observed over longer times. These experiments thus demonstrate the homeostasis in global activity maintained by the neuronal networks while being able to learn and adapt to stimuli through specific connectivity paths. We show this at a spatial scale of a neuronal network and a temporal period of 4 hrs after repetitive stimuli. First, we show that response of the individual neurons correlate with spontaneous activity of the network. Then we define a state of the network from the spontaneous activity recorded from the electrodes using principle component analysis. We then show how this state gets affected by theta burst stimuli along with persistent long term changes along specific connectivity paths.

## Methods

### Neuronal Culture

All animal experiments were performed in accordance with guidelines, rules and regulations of the Institutional Animal Ethics Committee (IAEC) for animal experiments of the Indian Institute of Science, Bangalore, India constituted as per article number 13 of the CPCSEA (Committee for the purpose of Control and Supervision of Experiments on Animals, http://cpcsea.nic.in) rules, laid down by Government of India.

Neuronal culture growth and maintenance was done using standard procedures^14,15^. Briefly, dissociated neuronal cell cultures were prepared from hippocampus of 0–2 day old Wistar rat pups on 120 multi-electrode array (MEA) dishes from MultiChannel Systems, Germany. Micro-dissected hippocampus was digested in papain solution and plated on electrode region of the MEA coated with laminin. The dishes were flooded with 1 ml of medium after the cells had adhered to the substrate, and stored with ethylene-propylene membrane lids in a 65% RH incubator at 37 °C, 5% CO_2_.

We used antibiotic/antimycotic drugs to control contamination. Feedings consisted of 50% medium replacement twice per week. The medium was used with glial conditioning (ara-C) after 7 days.

The culture dish was placed in a separate incubator which maintained an ambient of 5% CO_2_ at 37 °C while doing recordings and stimulations.

### Recording and Stimulation

We used MEA-2100 System from MultiChannel Systems, Germany© for recording from and stimulating the cultures grown on the MEA. The hardware was used to record signals from 120 channels simultaneously at 50 kHz and to generate stimulus pulses at all electrodes under software control.

### Measurements

The data was acquired from the device using MATLAB. Spike detection was done on the acquired data for further processing. This required filtering, artifact suppression and appropriate threshold crossing detection which was done on-line using MATLAB. Threshold for each electrode was estimated as 5x standard deviation (estimated using median values) and was applied on the absolute value of the signal^14,16^.

For electrical stimulation we chose the parameters which have been shown to be effective in previous studies^14,16,17^. For each stimulus we used a bi-phasic voltage pulse of amplitude 500 mV and a pulse width of 500 µs in each phase.

### Principle Component Analysis

**Figure 1:**
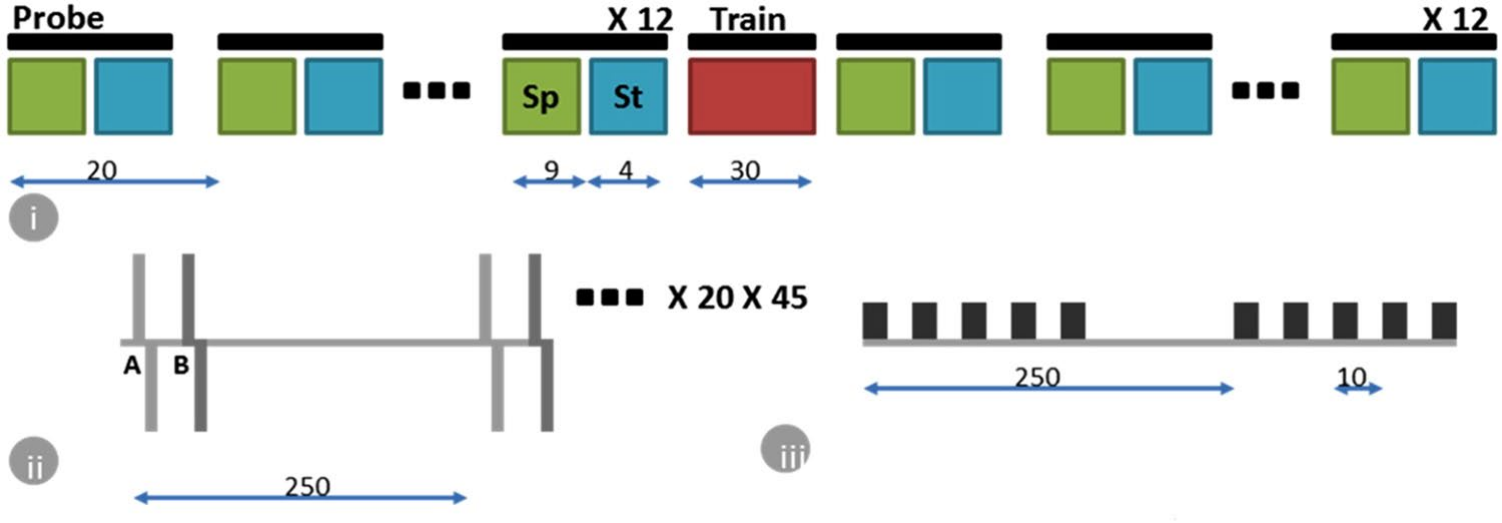
Figure shows the details of the experimental protocol in an 8.5 hr session on a day. (i) 12 monitoring sequences, each separated by 20 min, capture the state of the neuronal network before applying a training sequence (Train) for 30 min. This is followed by another set of 12 monitoring sequences to monitor the evolution of the network activity in a 4 hr window after training. Each monitoring sequence consists of a 9 min recording of spontaneous activity (Sp) and 4 min recording of stimulus evoked response (St) using probe sequences consisting of spatio-temporal stimulus patterns. This allows us to investigate, both the ongoing spontaneous activity in the network, as well as response of the network to stimuli during the same period. (ii) The spatio-temporal stimulus pattern consists of paired stimulus at 2 electrodes A and B (from a possible set of 8). The stimulus waveform at each electrode is a 500 µV biphasic voltage pulse with each phase lasting 500 µs. The time delay between end of first pulse and start of next is 500 µs. The time delay between the paired stimulus at the two electrodes is one of 0.5 ms or 3 ms. A probe sequence consists of applying 20 different spatio-temporal stimulus patterns (from a possible set of 56), applied 45 times, and each application has the 20 patterns in a random order. A delay of 250 ms is used between each stimulus pattern. (iii) The training sequence consists of repeated stimulation using one of the spatio-temporal stimulus pattern at high frequency (10 ms/100 Hz) (theta burst stimulation), repeated every 250 ms for 30 min.

Principle Component Analysis (PCA) is a statistical method that uses orthogonal transformation to convert data of a (possibly correlated) set of components into another set of linearly uncorrelated components, such that the variance of the data along each of the transformed components is in a decreasing order. In neuronal signal analysis, firing rates from a large number of neurons is reduced to a smaller number of components which account for most variance in data. These components show dimensions of information embedded in the firing pattern and might reflect the functional association between the neurons in the ensemble. We explain how we used the spontaneous activity recorded from the culture (see experimental protocol in Fig. 1) in the section on output decoding. To calculate PCA on the spontaneous activity, we proceed as follows.

Let $$x(k)=[\begin{array}{cc}{x}_{k1}\ldots  & {x}_{kp}\end{array}]$$ be the kth observation with p components. Then under PCA, we find the matrix *W* (of size *p* × *p*) which transforms *x*(*k*) to *y*(*k*) as:

$$y(k)=x(k)\ast W$$

If there are n observed data points *x*(1), … *x*(*n*) (each of dimension *p*), then

$$X=[\begin{array}{c}x(1)\\ \ldots \\ x(n)\end{array}]$$

$$Y=[\begin{array}{c}y(1)\\ \ldots \\ y(n)\end{array}]$$

and using PCA technique, we find W so that

$$Y=XW$$

and with the property that the variance of the first column of $$Y=\,{[\begin{array}{ccc}{y}_{11} & \ldots  & {y}_{n1}\end{array}]}^{T}$$ is the largest, followed by that of the second, third etc. columns in decreasing order. If only the first ‘l’ columns have significant variance, and if l ≪p, then it becomes easier to analyze/visualize the data in the transformed space. For our analysis, we use the data from the spontaneous network activity, recorded prior to the training (see Fig. 1), to obtain the transformation matrix *W*. Post-training, we use the same *W* to transform the post-training data to enable comparison of correlations pre and post training and thus make some inference about changes in the network state.

## Experimental Protocols

### Input Patterns

Spatio-temporal stimulus patterns were created by selecting appropriate electrodes for stimulation with a temporal delay amongst stimulation pulses at these electrodes^14^. Eight electrodes which evoked maximum response from the culture upon applying stimulation through each of them individually, were selected as candidate electrodes and were labeled A,B,C,D,E,F,G,H. These gave us eight spatially distinct sites for stimulation. Spatio-temporal stimulus patterns were created by pairing any two of these electrodes and applying stimulus at each with a short time delay of either 0.5 ms or 3 ms, resulting in 56 different stimulus patterns (AB, BA, AC…). To monitor the stimulus evoked response to the stimulus pattern, we apply a probe sequence consisting of an application of 20 of these stimulus patterns, 45 times, with a random order of the patterns within each application and with an inter-pattern time interval of 250 ms, as described in Fig. 1.

Each experiment was performed over a period of 8.5 hrs as shown in Fig. 1. The network activity was monitored for 4 hrs, followed by a training session of 30 min. This was followed by monitoring of the network activity for 4 hrs.

Each 4 hr monitoring period consisted of 12 monitoring sequences, each separated by 20 minutes. Each monitoring sequence consisted of monitoring the resting state activity (RSA) of the network for 9 minutes using the spontaneous activity recorded at the electrodes and a 4 minute interval where the network was stimulated using aforementioned probe sequences and responses recorded.

The training session consisted of theta burst stimuli created by presenting one of the spatio-temporal stimulus patterns (Stimulation at a pair of electrodes with a time delay of 0.5 ms). A burst of 5 repetitions of the stimulus pattern spaced at 10 ms (100 Hz) is repeated every 250 ms for 30 minutes.

### Output decoding

We defined the output vector from the culture for each pattern as a 120 element binary vector indicating occurrence of a spike in a 100 ms post stimulus window.

1 $${X}_{k}^{j}=[s{1}_{k}^{j}\,s{2}_{k}^{j}\,s{3}_{k}^{j}\,\cdots \,s{120}_{k}^{j}]$$

${X}_{k}^{j}$ is the output pattern for the culture for the *k*^*th*^ presentation of input pattern *j*. Here $s{M}_{k}^{j}$ is the spike occurrence indicator for electrode *M* and is defined as $s{M}_{k}^{j}=1$ if at least one spike occurs in the time window 5 *ms* to 100 *ms* after the *j*^*th*^ input pattern is presented to the culture *k*^*th*^ time.

For further analysis, we estimated the probability of spike occurrence ${{\rm{p}}}_{{\rm{M}}}^{{\rm{j}}}$ at electrode ‘M’ in response to stimulus pattern j as

2 $${{\rm{p}}}_{{\rm{M}}}^{{\rm{j}}}=\frac{{\sum }_{k=1}^{45}{{\rm{sM}}}_{{\rm{k}}}^{{\rm{j}}}}{45}$$

The spontaneous activity at an output electrode at time t, s_M_(t), was set to total number of spikes observed at electrode M during the spontaneous activity recording started at time t.

Spontaneous activity of the entire network, *SN*(*t*) at time *t* was then viewed as a vector of 120 elements with each element corresponding to one electrode and indicating the total number of spikes at that electrode.

3 $$SN(t)=[{{\rm{S}}}_{1}({\rm{t}})\,{{\rm{S}}}_{2}({\rm{t}})\,\ldots \,{{\rm{S}}}_{120}({\rm{t}})]$$

For an experiment over an 8 hr window, a total of 24 such vectors, capturing the spontaneous network activity was collected and grouped into pre and post training set.

4 $$S{N}_{pre}=[\begin{array}{c}SN(1)\\ \ldots \\ SN(12)\end{array}]$$

5 $$S{N}_{post}=[\begin{array}{c}SN(13)\\ \ldots \\ SN(24)\end{array}]$$

Similarly, for a stimulus pattern *j*, the evolution of response at time *t* can be captured as ${{\rm{p}}}_{{\rm{M}}}^{{\rm{j}}}({\rm{t}})$ and evolution of network response for this probe pattern *PN*^*j*^ can be captured as,

6 $$P{N}_{pre}^{j}=[\begin{array}{c}P{N}^{j}(1)\\ \ldots \\ P{N}^{j}(12)\end{array}]$$

7 $$P{N}_{post}^{j}=[\begin{array}{c}P{N}^{j}(13)\\ \ldots \\ P{N}^{j}(24)\end{array}]$$

where *PN*^*j*^(*t*) is the probe response of the network for probe j at time t at 120 electrodes

8 $$P{N}^{j}(t)=[{{\rm{p}}}_{1}^{{\rm{j}}}({\rm{t}}){\,{\rm{p}}}_{2}^{{\rm{j}}}({\rm{t}})\,\ldots \,{{\rm{p}}}_{120}^{{\rm{j}}}({\rm{t}})]$$

To understand the evolution of the network over time, we used Principle Component Analysis (PCA) as described in Methods section. This method has been previously used to study state dynamics from recordings of multi-unit activity^18^. The method identifies principal axes of variation in data, which are viewed as underlying state of the network modulating the activity at large number of multiple units. This general method has been proved useful in dimensionality reduction for viewing large-scale multi-dimensional data to understand important changes.

The steps performed for principal component analysis is summarized below

1. Principal component scores and the projection weights were first computed using the spontaneous activity collected before the training period. Mean value for each electrode before training was also computed.
  9 $$\langle W,\,{Y}_{pre}\rangle =PCA(S{N}_{pre})$$
  *where* W is a 120 × 120 matrix and
  $${Y}_{pre}=(S{N}_{pre}-{\mu }_{pre})\ast W$$
  and
  $${\mu }_{pre}=[\mu (1)\ldots \mu (12)]=[mean(SN(1))\ldots mean(SN(12))]$$
2. Spontaneous activity observed post training was projected onto the same components as identified using pre-training data.

10 $${Y}_{post}=(S{N}_{post}-{\mu }_{post})\,\ast \,W$$

where

$${\mu }_{post}=[mean(SN(13))\ldots \,mean(SN(24))]$$

We found that the first three columns Y_pre_(*,1), Y_pre_(*,2), Y_pre_(*,3) captured the major changes in the spontaneous activity observed in the network and hence were defined as state vectors for further analysis. This, along with the network probe response allowed us to see the evolution of the network over a long time scale and the effect of training protocols. The post training spontaneous data was projected using the *W* obtained from pre-training data to estimate if the internal state of the network changed.

## Results

The experiments were carried out with 3 cultures on different days *in vitro* (DIV 30–40). Detailed analysis is presented for one of these sessions (of the 11 sessions analyzed). Other trials show similar results.

**Figure 2:**
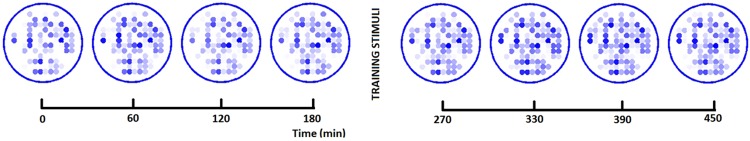
Response to a specific spatio-temporal stimuli changes over time. Each circle shows spatio-temporal response of the network recorded from 120 electrodes to a specific stimulus pattern over an 8.5 hr window at times indicated. The responses shown are obtained from analyzing the outputs from probe sequences that are 60 min apart. The intensity of each dot indicates the probability of observing a spike at an output electrode, in response to that specific spatio-temporal stimuli. It can be seen that at some electrodes, post training, the probability of a spike increases while for others it decreases.

**Figure 3:**
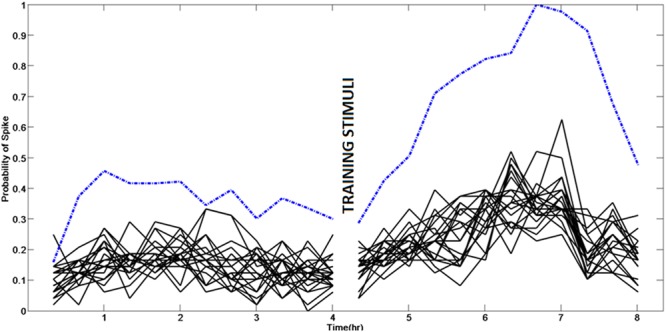
The probe response following stimulation with a training pattern at a particular output electrode to different probe patterns (black lines) is correlated with the spontaneous activity at that electrode (dashed blue line). Thus spontaneous activity recorded at an electrode could indicate its functional connectivity and current ability to respond to input stimuli. The spontaneous activity at 120 such electrodes vary differently. From this activity, we define state of the network in lower dimensions which could more concisely capture the overall network behavior and thus allow prediction of spontaneous activity and thereby response to inputs.

Figure 2 shows the response at 120 electrodes for a network over 8 hrs. One can see that the response to a probe changes over time. We recorded the resting state activity (RSA) at all the electrodes during the same experimental window. In Fig. 3, we show the stimulus response to different stimulus probes as well as RSA at a single electrode. We can see that the probe response correlates with spontaneous activity at that electrode. This indicates that spontaneous activity at every electrode could be a good predictor of changes in stimulus responses.

PCA was then used to extract the main components from the spontaneous firing rate data. There were three main components and these defined the network state.

**Figure 4:**
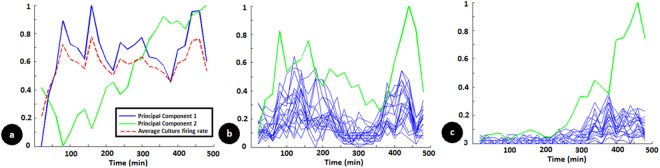
(**a**) Principle component analysis (PCA) of spontaneous activity evolution at multiple electrodes in the multi-electrode array. The solid lines indicate the first two principal components and the dashed red line indicates the average culture firing rate. (**b**,**c**) Spontaneous activity (green) and stimulus evoked responses (blue lines with different probe stimuli) at two different electrodes from the same multi-electrode dish used in (**a**). This shows how spontaneous activity at individual electrodes is reflected in the stimulus evoked responses. In (**b**), the spontaneous activity and the stimulus evoked response correlate and it is largely the reflection of the first principal component shown in a. In (**c**), the same correlation is observed between stimulus evoked and spontaneous activity but this largely reflects the second principal component shown in (**a**). It also shows how spontaneous activity at individual electrodes is different from the overall network average but can be explained by a linear combination of principal components.

Figure 4 captures the results of PCA in a recording session where network shows a continuous change. Figure 4a shows the evolution of average culture activity over time. It also shows the first two principal components extracted from the spontaneous activity at 120 electrodes. We can see that the first component largely captures the overall network response while the second principal component shows a gradual increase over time thus capturing another underlying change happening in the network state. This now explains the changes at individual electrodes as shown in subsequent figures. In Fig. 4b, the spontaneous activity as well as probe sequence response largely follows the first principal component whereas in Fig. 4c, at a different electrode it largely follows component 2. Other electrodes follow a combination of these two components. Thus we can see that the overall changes happening at 120 electrodes are different for different electrodes but can be captured by a few internal states. These internal states also affect the probe responses and thus network patterns generated at 120 electrodes over time.

We then defined a 3 dimensional vector using the first three principal components, Y_pre_(*,1), Y_pre_(*,2), Y_pre_(*,3), and call it the internal state of the network. Next, we analyzed the changes in state of the network post training. We applied a training session consisting of bursts of stimuli (see Methods) to trigger changes in the network after 4 hrs of recording. We then calculate the state of the network post training by finding Y_post_(*,1), Y_post_(*,2), Y_post_(*,3) using the same transformation matrix W, found using pre-training data (as explained in equations 9 and 10).

**Figure 5:**
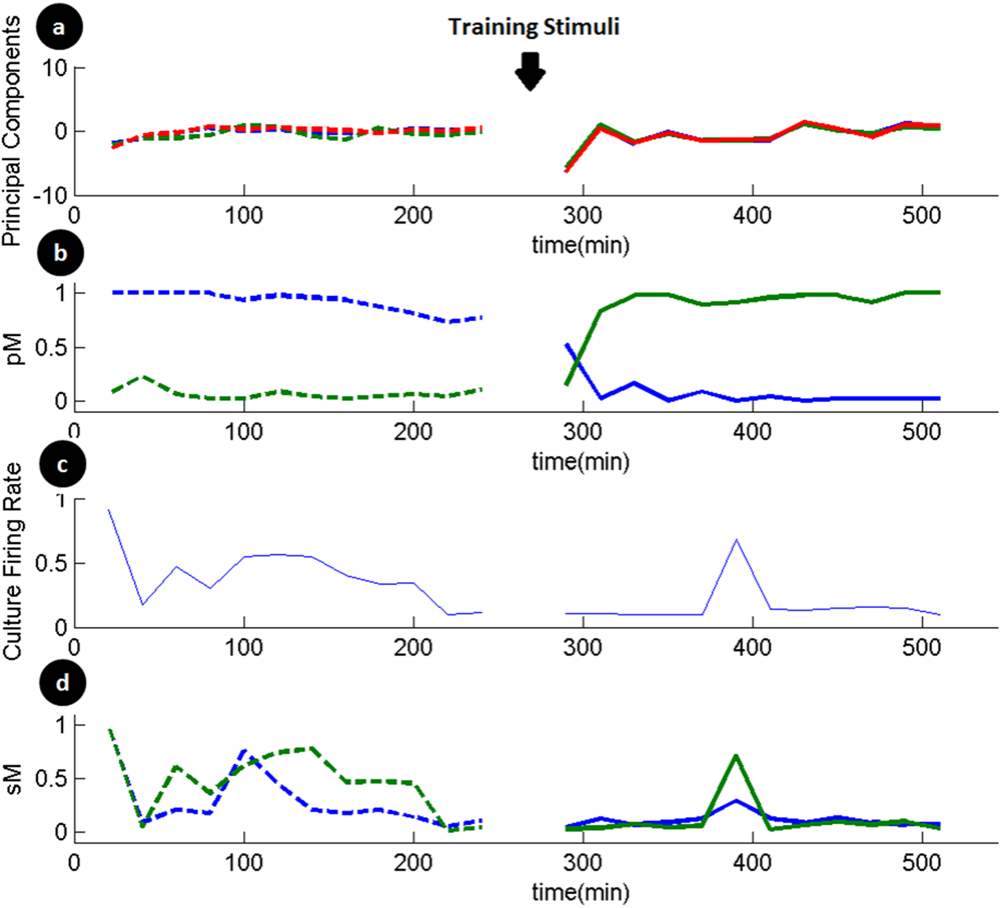
Homeostatic stability and plasticity at network and single neuronal levels following training stimuli. (**a**) Trace of three principal components of spontaneous network activity (blue: PC1, green: PC2, red: PC3). This shows that training protocol induces a temporary disturbance in state of the network which is restored after some time. (**b**) Change in probability of response (pM) to specific stimuli at two different electrodes. One electrode (Green) indicates a definite increase in probability of spike response while another (Blue) indicates a persistent decrease. (**c**) Average culture activity during the period of recording (Spontaneous activity at each electrode is normalized and the mean for all the electrodes is plotted). There is a gradual decrease in average culture activity over the course of the experiment which shows an evolving network. But, this is not significantly affected by training protocol. This is more relevant when one observes that there is a definite disturbance in the principal components extracted from spontaneous activity which shows that pattern of activity is disturbed while overall average being same. (**d**) Spontaneous firing rate at electrodes (sM) indicated in b with same color. Though there is a persistent shift in evoked firing due to training, it is not so significant in spontaneous firing rate which largely follows the overall network activity as in c.

In Fig. 5, we show the changes in state of the network as well as responses at some specific electrodes. The state of the network shows a temporary change after training and is restored after some time. We note that though the state components show a change, the mean culture activity remains the same as indicated in the figures. Next, we show probability of spike to two different stimulus patterns at two different electrodes. We conjecture that this is representative of the strength of the path between these electrodes. We can see a long lasting change in this strength which can result in either an increase or a decrease of the spiking probability of the electrode. This demonstrates that a spatio-temporal training pattern can cause a complex modification of the network response to stimulus. We also note that even though there is a definite change in the stimulus response at these electrodes, the spontaneous firing rate, as well as the network state seems to be largely unaffected by the burst training stimuli, thus suggesting local network modification.

## Discussion

We show that response of the neuronal network to stimuli is correlated with the spontaneous activity at that electrode as it varies over time (Fig. 3). We then defined a vector using the average firing rate at different electrodes in a 9 min window (Fig. 1) as it evolves over a period of 8 hrs (Eqn 4). Using PCA we then transform the spontaneous firing rate data into a lower dimensional space of 3 components which defines the network state space. We then study the state trajectory based on these principal components and found that as few as three components capture a significant amount of the overall change happening in the network. We used this low-dimensional subspace to denote the network state space and it allowed us to visualize the changes happening in the network.

We find that complex persistent changes can be brought about by theta burst stimuli at specific contact points along specific connectivity paths, even while the network maintains its overall homeostasis. This helps the network create rich, robust and adaptable population codes useful for context dependent input processing^16^.

### Resting State Activity reflects prior expectations of an internal model of the stimulus

The spontaneous activities sampled by the electrodes reflect the ongoing network dynamics. This is also referred to as resting state activity (RSA). Simultaneous recording of network activity using optical imaging coupled with single unit recording show the link between them^19^. The anatomical and physiological basis for this is the synaptic connections received by the neurons from the network^20^. With a higher ongoing activity, the membrane remains in a more depolarized state and the rate at which it would cross the threshold for spiking is higher. This also affects the stimulus response. This has been studied by simultaneously monitoring the network activity using multi electrode array and membrane potential using patch clamp^21^.

Our experiments also highlight this link between the ongoing resting state activity and the stimulus evoked responses (Fig. 3). It has been suggested that such a resting state activity reflects a prior expectation or the internal model for the stimuli which is gradually adapted during development^22^. It is hypothesized that such activity represents prior expectations about different stimuli the network would receive and make a probabilistic interpretation about the actual stimulus in the evoked response^22^. For instance, an electrode showing a high rate of firing would indicate that most of the stimuli would cause a higher response at that electrode. This in turn reflects an internal expectation of the neuronal culture about the stimuli, that is, most of the stimuli are such that a higher response would be elicited at the particular electrode. This view puts the network in a state of constantly interpreting the environment which when combined with actual stimuli generate the most probable representation of the input. Our results show that spontaneous activity at an electrode reflects the probability of the electrode showing a response to the stimulus.

### Network dynamics can be captured in a low dimensional space

Interestingly, only a few variables reflect the changes happening at large number of electrodes indicating that over time, the changes happening in the network are global in nature than being specific at different locations of the network (Fig. 4). If the network state trajectories did not vary and if these were not identifiable in a low dimension, it would have indicated random changes in the neuronal activity at different electrodes.

Such an internal state coordinating the processing of different cell assemblies have been considered important in higher cognitive functions^23^ (autobiographical memory, self-referential thought, and mind wandering). These have been observed using various imaging studies (EEG, MEG, fMRI, PET).

### Networks can generate complex population codes which can be changed by training

Neuronal cultures have a rich repertoire of population codes for stimulus patterns. Consider the response of two electrodes (Blue(E1) and Green(E2)) in Fig. 5 for the same stimulus probe. In the initial state, E1 has a high response and E2 has a low response indicating a population code {1,0}. In the final state, for the same stimulus, E1 has a low response and E2 has a high response showing a population code {0,1}.

Now consider the response at a different electrode (E3) for two different probes {B,A,3}(P1) and {B,A,4}(P2) in Suppl. Figure 1. These have same spatial location (Electrodes A and B) and differ only in the temporal structure. Initially, both these probes have same response at E3 and track each other as the network state changes. However, after training, P1 evokes a high response at E3 while P2 evokes a low response.

With a large number of such neurons, which respond differently to different input stimuli, the neuronal culture generates distinct population codes which are modulated by training stimuli (Fig. 3).

A global state coordinating the overall behavior of the neuronal network is indicative of an overall modulation of stimulus responses. However, this does not mean that all stimulus responses would be identical and largely dependent on internal states. We have shown previously that such cultures have a rich response behavior to stimulus probes where distinct population codes are generated for different stimuli. The neuronal cultures can generate linearly separable population codes for spatio-temporal patterns of input^14^. Such patterns show a coarse-conjunctive coding of inputs which result in a very robust population code suitable for classification^16^. Here we show that such properties are dynamically tunable based on the state of the network and training. Together, we can conclude that random neuronal networks have very rich processing capabilities which are state dependent.

### Theta burst stimuli cause a temporary change in network state which is restored after some time

Short term suppression in spontaneous activity has been reported in various studies^24^ and is thought to operate by clamping of internal dynamics from a chaotic state to fixed states^13^. Different models suggest abrupt switch in network state such as between sleeping and awake^25–27^. Plasticity in spontaneous activity structure has been reported in many studies. This has been observed immediately after stimulus^28^ and on training in regions of visual cortex^29^. These studies focus on periods immediately after stimulus inputs^30^.

Long-term changes in resting state activity or network states have not been adequately studied in cultures. Neuronal cultures have been used to study training and learning in neuronal networks^8,9^ where, the training stimuli were applied to change the response of the network to a probe pattern in a desired direction. However, these methods have not been used to study network states, their evolution over time, and its effect on processing of stimuli or long-term persistent changes. Changes in correlation are reported in a short time window immediately after training^11^. Long term effects of disturbances have been studied using cultures on multi electrode arrays^31^. Such networks were treated with drugs which resulted in suppression of activity which was later restored by synaptic adaptations after few days. However, these lead to change in firing patterns of the network which would imply a loss of memory even though activity level in the network is restored.

Maintaining this background activity is found to be important, disruptions of which can lead to neurological disorders^6^. Several mechanisms have been proposed for this homeostatic behavior. These include activity-dependent regulation of intrinsic neuronal firing^32,33^, Synaptic scaling^10,34^, the balancing of excitation and inhibition^35,36^, compensatory changes in synapse number^37,38^, mechanisms that adjust the ease of inducing LTP and LTD^39^ and homeostatic regulation of intrinsic excitability^40,41^. However, functional demonstrations of such mechanisms are lacking.

In Fig. 5, we show that after repetitive stimuli, the network state is disturbed. However this is restored after a brief period. We also note that the average culture firing rate is not affected drastically during this period. Thus theta-burst stimuli cause a short term network wide change in network activity which gets restored after some time. This shows homeostasis at spatial level of a neuronal network and temporal period of hours along specific connectivity paths. This ability of the network to maintain homeostasis is crucial for its stability.

Previous studies have shown that the global homeostasis in the overall firing rate of the network could be due to the regulation of maximal firing rate of the neurons^42,43^.

### Theta burst stimuli leave a long-term trace of memory in some of the paths

While maintaining stability, it is also important that traces of the repetitive action are persistent along specific functional connectivity paths in the network. This forms the basis of learning and memory.

In Fig. 5, we show an electrode which shows an increase in probability of firing and another electrode which shows a decrease in firing probability. Such pathways specific changes have been reported previously and were associated with pre-training correlation of activity^11^. Together, with other such electrodes, they cause a change in population code for different stimuli. These changes gradually evolve in response to a training protocol and are persistent. This indicates that such changes are triggered by the training protocol, takes time to happen and are more permanent in nature as opposed to temporary fluctuations due to Short Term Plasticity. We conclude that burst protocol triggers consistent changes and would be due to synaptic and neural plasticity mechanisms. It is also possible that the strongest connections in the network respond to theta burst stimuli by further increasing their strength relative to other connections in the network. This mechanism might, in all likelihood, preserve connections that are most informative and relevant to the overall network^31^.

We note that while there is a persistent change in response to specific stimuli, the overall firing rate of both the neurons is largely unchanged. This indicates some form of homeostasis even at single neuron level while still being responsive to theta burst stimulus. The average firing rate of the neuron may be due to its responses to overall stimuli received from rest of the culture and it remaining stable would indicate appropriate adaptation of synapses to maintain it, in spite of changes for learning.

### The Neural Plasticity mechanisms observed are in a timescale of ~30 Mins

We found that the mechanisms that create the network changes operate at a timescale of mins-hrs indicating gene expression and protein synthesis machinery to be triggered by the training protocol lasting for 30 minutes and causing functional connectivity changes through appropriate receptors. Activity-induced increases in translation at the synapse and dendrites can indeed be very rapid as shown in *in vitro* studies and increased mRNA translation can be initiated within minutes after stimulation^44,45^.

### The Neural Plasticity mechanisms operate at a dendritic level than at independent synapses

Recent computational studies suggest the possibility of learning happening in neurons/dendrites(nodes) as opposed to synapses(links) in the network^46^. We find that training stimuli change the spontaneous activity response of the neurons as well as the stimulus evoked responses in similar way. In Fig. 3, the spontaneous activity as well as the stimulus evoked responses post training follows a similar trend at an electrode. In Fig. 4b,c, a similar response is observed at two different electrodes. Considering that the stimulus probes are applied at spatially distinct electrodes and spontaneous inputs from the culture to a neuron is expected to converge from different paths, all the inputs following a similar trend suggest a more local modification of the properties than a similar change at all the synapses.

In Fig. 5, we show neurons which exhibit a change in stimulus evoked response different from the spontaneous activity of the neurons. We also note that it is these links which retain the specific probe learnings over a long time. We expect that these nodes (dendrites) do not receive significant spontaneous activity from the network to cause a change in the learning that happened due to training stimuli. This could explain the observed phenomena why most of the learning in neuronal cultures is not retained for long time as these get modified due to spontaneous activity.

## Conclusion

In conclusion, we have shown that neuronal networks maintain homeostatic regulation of activity, in spite of disturbances caused by repetitive stimuli while also retaining the property of persistent change along specific paths. These networks can do effective processing of complex spatio-temporal stimuli which is modulated by internal state of the network. A link between the network state estimated from spontaneous activity and response to stimuli has been brought out. We have then shown that theta-burst stimuli can cause changes in spatio-temporal pattern of response to probe stimulus pattern without significantly changing overall state of the network. The network maintains an overall consistency in its state though it is temporarily disturbed by theta-burst stimuli. Such homeostasis mechanisms will allow it to maintain consistency as well as adaptability. Our experiments show homeostatic maintenance of firing rate at individual neurons in a network, while also being adaptable to stimuli at specific paths.

## Electronic supplementary material

Supplementary Information

## Data Availability

The datasets generated during and/or analyzed during the current study are available from the corresponding author on reasonable request.
