# Supplementary material for: Random Neuronal Networks show homeostatic regulation of global activity while showing persistent changes in specific connectivity paths to theta burst stimuli
Source: Sci Rep. 2018 Nov 8;8:16568. doi: 10.1038/s41598-018-34634-x (PMC6224599; doi:10.1038/s41598-018-34634-x)
Supplement: Supplementary file 1 — Supplementary Information [file 41598_2018_34634_MOESM1_ESM.docx]

Random Neuronal Networks show homeostatic regulation of global activity while showing persistent changes in specific connectivity paths to theta burst stimuli

Jude Baby George^a^, Grace Mathew Abraham^a^, Bharadwaj Amrutur^b^, *Sujit Kumar Sikdar^c^

*^a^Center for Nanosicence and Engineering, IISc Bangalore ^b^Robert Bosch Center for Cyber-Physical Systems and Department of Electrical Communications Engineering, IISc Bangalore ^c^Molecular Biophysics Unit, IISc Bangalore*


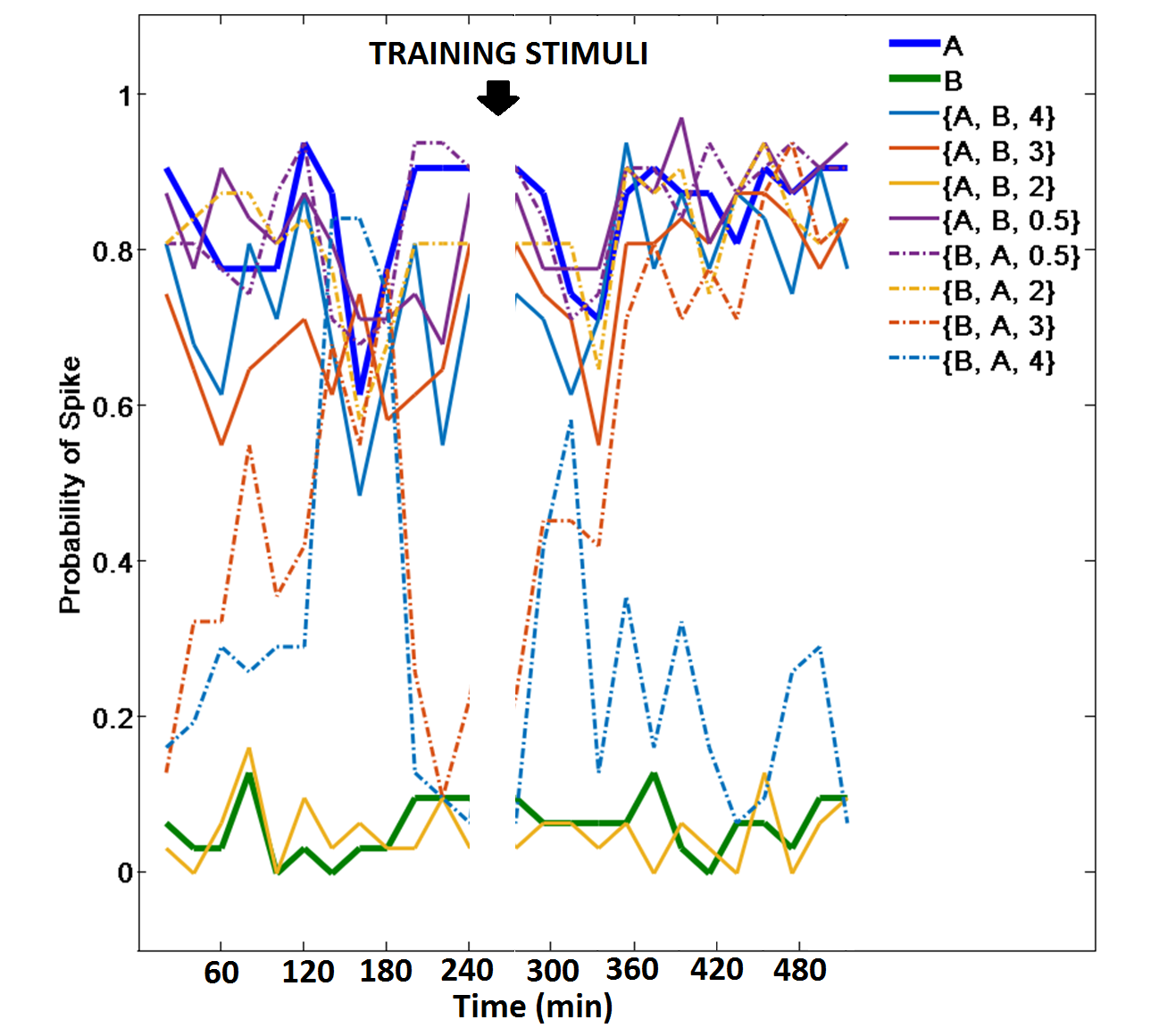


Supplementary Fig. 1: Time dependent changes in the responses to different spatio-temporal stimulus patterns (P) at an electrode in the multi-electrode array reflecting convergence of inputs *via* different paths. Here, probability of response at an output electrode M for different input stimuli are shown that were constructed using same input electrodes {A,B} with different time delays (in ms). It can be seen that a definite change is visible for the pattern {B,A,3} while there is no significant change for the pattern {A,B,0.5}. This suggests that network response changes could be very selective to different spatio-temporal input stimuli which can be attributed to activation of different functional connectivity paths. The inherent variability in the observed responses may explain the difficulty in constructing training patterns for specific changes in network response.
